# Combining administrative data feedback, reflection and action planning to engage primary care professionals in quality improvement: qualitative assessment of short term program outcomes

**DOI:** 10.1186/s12913-015-1056-0

**Published:** 2015-09-18

**Authors:** Brigitte Vachon, Bruno Désorcy, Isabelle Gaboury, Michel Camirand, Jean Rodrigue, Louise Quesnel, Claude Guimond, Martin Labelle, Ai-Thuy Huynh, Jeremy Grimshaw

**Affiliations:** School of Rehabilitation, Faculty of Medicine, Université de Montréal, 7077 Park Avenue, Montreal, Quebec H3N 1X7 Canada; Agence de la santé et des services sociaux de la Montérégie, 1255 Beauregard Street, Longueuil, Quebec J4K 2M3 Canada; Faculty of medicine, Université de Sherbrooke, Pavillon Gérald-La Salle, 3001, 12e avenue Nord, Sherbrooke, Quebec J1H 5N4 Canada; Centre de santé Sutton, 33 Principale St South, Sutton, Quebec J0E 2K0 Canada; Collège des médecins du Québec, 2170, boulevard René-Lévesque Ouest, Montreal, Quebec H3H 2T8 Canada; Fédération des médecins omnipraticiens du Québec, 3500 boul. de Maisonneuve Ouest, bureau 2000, Westmount, Quebec H3Z 3C1 Canada; Centre de recherche de l’Institut universitaire en santé mentale de Montréal, 7331, rue Hochelaga, Montreal, Quebec H1N 3V2 Canada; Centre for Practice-Changing Research, Ottawa Hospital Research Institute, The Ottawa Hospital – General Campus, 501 Smyth Road, Box 711, Ottawa, Ontario K1H 8L6 Canada

**Keywords:** Quality improvement, Primary care, Interprofessional collaboration, Continuing professional development, Chronic disease management, Administrative data, Reflection, Action planning

## Abstract

**Background:**

Improving primary care for chronic disease management requires a coherent, integrated approach to quality improvement. Evidence in the continuing professional development (CPD) field suggests the importance of using strategies such as feedback delivery, reflective practice and action planning to facilitate recognition of gaps and service improvement needs. Our study explored the outcomes of a CPD intervention, named the COMPAS Project, which consists of a three-hour workshop composed of three main activities: feedback, critical reflection and action planning. The feedback intervention is delivered face-to-face and presents performance indicators extracted from clinical-administrative databases. This aim of this study was to assess the short term outcomes of this intervention to engage primary care professional in continuous quality improvement (QI).

**Methods:**

In order to develop an understanding of our intervention and of its short term outcomes, a program evaluation approach was used. Ten COMPAS workshops on diabetes management were directly observed and qualitative data was collected to assess the intervention short term outcomes. Data from both sources were combined to describe the characteristics of action plans developed by professionals. Two independent coders analysed the content of these plans to assess if they promoted engagement in QI and interprofessional collaboration.

**Results:**

During the ten workshops held, 26 interprofessional work teams were formed. Twenty-two of them developed a QI project they could implement themselves and that targeted aspects of their own practice they perceived in need of change. Most frequently prioritized strategies for change were improvement of systematic clientele follow-up, medication compliance, care pathway and support to improve adoption of healthier life habits. Twenty-one out of 22 action plans were found to target some level of improvement of interprofessional collaboration in primary care.

**Discussion:**

Our study results demonstrate that the COMPAS intervention enabled professionals to target priorities for practice improvements and to develop action plans that promote interprofessional collaboration. The COMPAS intervention aims to increase capability for continuous QI, readiness to implement process of care changes and team shared goals but available resources, climate and culture for change and leadership, are also important required conditions to successfully implement these practice changes.

**Conclusion:**

We think that the proposed approach can be very useful to support and engage primary care professionals in the planning stage of quality improvement projects since it combines key successful ingredients: feedback, reflection and planning of action.

**Electronic supplementary material:**

The online version of this article (doi:10.1186/s12913-015-1056-0) contains supplementary material, which is available to authorized users.

## Background

Improving primary care for chronic disease management requires a coherent, integrated approach to quality improvement (QI). Developed in the United States, the Chronic Care Model is the best described and widely-known model for chronic disease management [1–3]. The actions it promotes are intended to generate proactive, organized healthcare teams interacting with informed active patients. Generally speaking, the review articles point out that implementation of this primary care model is associated with better health outcomes for people with chronic diseases [1, 4–6]. A meta-analysis suggests that successful implementation of at least one characteristic of the Chronic Care Model is associated with the improvement of healthcare processes and health outcomes for people with asthma, diabetes, heart failure, and depression [7].

Shared care and interprofessional collaboration (IPC) are some of the key components of this model and of chronic care management [8–10]. IPC in primary care can be defined as « an integrative cooperation of different health professionals, blending complementary competence and skills, to the benefit of the patient, making possible the best use of resources in a primary care setting » [11, 12]. However, supporting the development of IPC in primary care remains challenging [11, 13]. One barrier is health professionals’ perception that they already work as a team, are competent to do so, and know each other’s roles and skills. Yet studies investigating this concept in primary care often describe both the lack of knowledge and understanding of coworkers’ roles displayed by these professionals and their lack of skills for effective interdisciplinary teamwork [14]. Mobilizing and engaging professionals in a shared service-quality improvement process require strategies that foster practice changes.

Evidence in the continuing professional development (CPD) field suggests the importance of using strategies such as feedback delivery [15] and reflective practice [16, 17] to facilitate recognition of gaps and service improvement needs. In a recent systematic Cochrane review [18], the effect of using audits and feedback was found to vary widely across the included studies, and the quality of the evidence was moderate. Feedback was found to be most effective when it reports greater gaps in health professionals’ performance, is provided more than once (both verbally and in writing), and includes targets and an action plan. One cost-effective way to provide feedback to primary healthcare professionals is to use administrative health data readily available for an entire population [19, 20]. As mentioned by Katz and collaborators [19], a key strength of this approach is the completeness of the data. The use of the indicators available in these databases has been found to facilitate comparisons among practices over time and against standards, to promote accountability and to support the identification of unacceptable levels of performance. However, administrative health data has its limitation since it is not collected for research purposes, but for billing purposes [19].

The literature also reveals growing interest in the use of action planning, which capitalizes on health professionals’ intention to change and transposes it into concrete action that will bring about change. Planning is a prospective self-regulatory strategy, a mental representation allowing a concrete response to be linked to future situations. It depends on and serves the purpose of a person’s specific intention [21]. The development of the action plan promotes the initiation of action by specifying the following elements [21, 22]: what do you want to change?; how do you want to bring about this change?; who will be responsible?; by when will the change be implemented?; what resources (outputs) are needed?; and what outcomes do you wish to produce? The expected outcome of the action planning process is the movement of a group towards a shared vision or goal. According to O’Neal [23], effective action planning can be indicative of an effective workplace culture where individuals and teams take responsibility for action and quality.

Our study explored the outcomes of a CPD intervention, named the COMPAS Project (COMPAS stands for collective for best practices and improvement in healthcare and services in family practice), which aims at engaging front-line clinicians in the QI of services they deliver to persons with chronic diseases. It was implemented in Quebec’s Montérégie region (Canada). The program impact theory of this intervention was described in depth in a previous published article [24] and was recently cited in example as a well-articulated program theory [25]. The basic assumptions underlying the COMPAS intervention is that healthcare professionals are individuals who are absorbed in their everyday practice and who lack the time and opportunities to self-evaluate and self-monitor their practices [24]. To change and improve their practice, these professionals are required to receive feedback, recognize gaps between actual and recommended practice, identify QI goals and also plan and implement strategies to improve care and patients’ health. The COMPAS intervention consists of a three-hour workshop composed of three main activities: feedback, critical reflection and action planning. The feedback intervention is delivered face-to-face. It presents performance indicators extracted from clinical-administrative databases. The aim of this study was to assess the short term outcomes of this intervention. More specifically, we evaluated whether combining feedback, critical reflection and action planning allowed primary care professionals working in the same community to 1) set cooperative and mutual practice improvement goals and 2) develop action plans that led to implementation of cooperative practice changes.

## Methods

In order to develop an understanding of our intervention and of its short term outcomes, the program evaluation approach proposed by Rossi, Lipsey and Freeman [26] was used. This approach allows for the evaluation of complex interventions implemented in a community setting where researchers have little control over characteristics of the participants, program, and organizational contexts. Rossi et al. [26] stress the importance of explicitly detailing a program’s action mechanisms by describing its aim and expected outcomes and the activities required to attain these outcomes. To assess a program impact theory, evaluators can conduct observations that will provide a reality check of the theory and assess if expected outcomes are realistically attainable. Between 2010 and 2012, ten COMPAS workshops on diabetes management were offered in most health and social service centres in Quebec’s Montérégie region. These workshops were directly observed and qualitative data was collected to assess the intervention short term outcomes. The study protocol was approved by the Research Ethics Committee of the Ottawa Hospital Research Institute. Participants were all informed, at the beginning of the workshop, that data was collected by the research team to document the intervention and its short term outcomes and that confidentiality of all participants would be respected.

### Description of the intervention

The COMPAS intervention is based on a reflective learning approach and takes the form of CPD workshops that target a specific chronic health problem [24]. The first series of workshops, offered in 2010–2012, targeted diabetes. It is designed to be offered to 20 to 25 professionals working in the same geographic area, thus serving the same population. Each workshop is led by two facilitators (a family physician and a community pharmacist or a nurse) who attended a half-day training session on the COMPAS intervention. The training took the form of a simulated COMPAS workshop where facilitators were also informed on how to facilitate small interprofesional learning groups [27]. The workshops’ specific objectives were to help participants: (1) develop a shared vision of their team performance; (2) develop a common understanding of performance gaps; (3) collaboratively identify one QI goal, and (4) collaboratively plan a practice change.

The workshops revolved around three main activities: receiving feedback on current practices, participating in a collaborative reflection process to identify service-quality improvement priorities, and developing an action plan. The feedback intervention involved presenting regional data extracted from administrative databases to the participants, as well as a set of relevant indicators to help them analyze their actual practice [24]. Participants were asked to form small working groups of 5 to 6 professionals combining different disciplines. Clinicians usually working together, for example from the same family medicine group, were asked to team up. They were instructed to critically discuss the data by answering the questions presented in Table 1, explain observed gaps in practices, and reflect on their respective roles and level of IPC. They then developed an action plan which targeted one specific QI goal using the provided template (Additional file 1) and described a service-quality improvement project they could implement in their practice setting. Both facilitators provided support to the small learning groups by answering their questions and making sure they first focused on practice gaps instead of jumping to solutions. Each activity (feedback, reflection, and action planning) was followed by a discussion in plenary which provided feedback to the small learning groups to pursue their reflective work.

**Table 1:** Process of developing an action plan in subgroups

Performance analysis:
- Which characteristics of patients with diabetes in your subregion attract your attention?
- What attracts your attention in terms of the use made of health services? (e.g. frequency of hospitalizations and emergency visits, frequency of visits to family physicians or specialists)
Identification of priority targets for change:
- What elements do you think should be targeted to improve the care delivered to patients with diabetes in your subregion? Identify one priority.
Interprofessional collaboration component:
- How can interprofessional collaboration help you attain this (these) objective(s)? Does this collaboration exist in your organization?

### Data collection and analysis

Two strategies were used to collect data: direct observation of the workshops and analysis of the participants’ action plans. The study’s principal investigator attended each workshop and took notes on the intervention and the action plans developed. The documents completed by the participants during their development of action plans in small groups were collected at the end of each workshop. The data from both sources were combined to describe the characteristics of each action plan and then analyzed for the purpose of answering the following questions: (1) what objective is targeted by the action plan?; (2) what type of diabetes prevention is targeted by the action plan?; (3) does the action plan engage the professionals in a process of continuous practice improvement?; and (4) does the action plan target the improvement of IPC in primary care? Data was coded by two individual coders (BV and ATH). A list of the targeted objectives was developed, categories were created and plans were classified by the two coders according to their main goal. In order to determine if action plans engaged or not primary care professionals themselves in QI, both coders evaluated if participants targeted an action they could realistically carry out or if their action required to be mainly carried out by someone else from their organization. Finally, we assessed if the action plans targeted improvement of IPC in primary care. Criteria used by both coders were: 1) professionals from at least two disciplines are targeted by the proposed change and 2) the proposed change is affecting how these professionals communicate together or share responsibility in the management of chronic diseases. Based on a framework developed by Careau and collaborators [28, 29], action plans were also classified according to what type of collaborative practice is expected to be achieved by the proposed change (Table2). The framework describes four types of collaborative practice which vary according to the level of interaction that exists between professionals from different disciplines. This classification was also independently coded by another investigator on our team (IG) who has an expertise in using this framework. Disagreements between coders were discussed to achieve consensus. When an action plan was considered to possibly target two types of collaboration, it was classified in the lower collaborative level.

**Table 2:** Types of interprofessional collaboration practice based on the framework developed by Careau and collaborators [28]

Types of IP collaborative practices	Definition
Parallel practice	Parallel interprofessional practice is characterized by a situation where a professional comes into contact with at least one professional from another discipline to inform or become informed about the services he delivers to the same person, family or community. Interactions between professionals are minimal or absent (ex: sharing reports and progress notes contained a patient file).
Consultation/reference practice	Consultation/reference practice is related to the intention to exchange and share information with at least one professional from another discipline. It involves recognition of one’s own expertise and limits and expertise and role of professionals from other disciplines. Interactions remain few and sporadic and professionals continue to work in parallel (ex: referral to another professional, consultation, assessment and treatment of a specific need).
Concerted practice	Concerted practice is based on the intention to plan and especially organize care and services in order to meet the biopsychosocial needs of a person, family or community. It aims to agree on disciplinary objectives and coordinate services provided by multiple professionals. The interaction is moderate and bidirectional. This type of collaborative practice is qualified as “multidisplinary”.
Shared healthcare practice	Shared healthcare practice involves shared decision-making and setting of common objectives and actions between professionals and the person, family or community. Interactions between professionals and the patient are necessarily more intense in this type of practice (interdependence and sharing of responsibilities). This type of collaborative practice is qualified as “interdisciplinary”.

This exploratory study did not initially provide for longer-term outcomes, making it impossible to formally document whether the majority of the action plans were actually implemented in each practice setting following the intervention. However, our team was informed of the implementation of a few action plans. To illustrate in more details how the COMPAS intervention led to implementation of cooperative practice change, two QI projects of different natures and scope are described.

## Results

Approximately 20 primary healthcare professionals (family physicians, nurses, community pharmacists, and nutritionists) participated in each workshop. During the ten workshops held, 26 interprofessional small working groups were formed and 22 of them developed action plans. Four groups were unable to develop a clear plan by the end of the workshop. Notes taken during workshop observation confirmed that grouping of participants who were unaccustomed to working together and practiced in different settings experienced more difficulty with action planning. The process appeared easier for work teams comprising at least a few professionals accustomed to working together and treating the same patients. Table 3 presents the main characteristics of the 22 action plans developed.

**Table 3:** Characteristics of the developed action plans (*n* = 22)

Type of prevention:	
- Primary^a^	1 (4.5 %)
- Secondary^b^	20 (91 %)
- Primary and secondary	1 (4.5 %)
Objectives of the action plan:	
- Improve systematic follow-up	5 (22.7 %)
- Improve medication compliance	5 (22.7 %)
- Improve service coordination/care pathway	4 (18.2 %)
- Encourage adoption of healthier lifestyle habits	3 (13,6 %)
- Improve retinopathy screening	2 (9,1 %)
- Increase use of multidisciplinary/community services	2 (9,1 %)
- Improve diabetes screening	1 (4,5 %)
Type of action plan:	
- Participant-owned^c^	20 (91 %)
- Delegated^d^	2 (9 %)
Targets interprofessional collaboration:	
- Yes	21 (95.5 %)
- No	1 (4.5 %)

^a^Primary prevention: Aimed at preventing diabetes. ^b^Secondary prevention: Aimed at preventing diabetes-related complications. ^c^Participant-owned action plan: A plan in which the actions required can be carried out by the project developers. ^d^Delegated action plan: A plan in which the actions required must be carried out by another party. In other words, the execution or not of the project is not the plan developers’ responsibility

The action plans targeted mainly secondary prevention, with the professionals favouring interventions involving patients who already had a diabetes diagnosis and focusing on actions for preventing diabetes-related complications. Only one team developed an action plan aimed at improving primary prevention. The most often identified priority was improvement of systematic clientele follow-up. As recommended in the clinical practice guidelines, the professionals frequently proposed developing a checklist to facilitate and systematize physician and nurse follow-up of all the complication risk factors. Some action plans aimed at greater pharmacist participation in this systematic follow-up by proposing the identification and development of effective means of communication between the pharmacy and family medicine group. Several action plans sought to improve interventions targeting changes in patients’ lifestyle habits and better self-management. For example, some family medicine groups considered it a priority to recruit a nutritionist or kinesiologist to their team or to facilitate referrals to such professionals in the community. Some action plans targeting better medication compliance were also developed and implied the need for cultivating better collaboration among the family medicine groups and community pharmacies involved to allow them to identify and take action with patients who stop taking their medication. Lastly, some action plans focused on improving service coordination by proposing the development, for example, of a community resources guide or a list of professionals working in the region, or a review of referral mechanisms and the content of education given to patients with diabetes in different clinical settings.

Twenty-one out of 22 action plans were found to target some level of improvement of IPC in primary care. One action plan was not described in sufficient details to allow classification. According to the framework developed by Careau and collaborators [28], a majority of the plans aimed at increasing consultation/reference collaborative practice (12/21) or parallel practice between at least two healthcare professionals (6/21). Two action plans were found to target a higher level of collaboration described as concerted collaborative practice. Notes taken during the workshop demonstrated that concerns of professionals in regards to collaboration remained at the level of findings better ways to share information and being aware of services available to increase referral.

It was therefore encouraging to observe, as anticipated, that the COMPAS intervention enabled front-line practitioners to develop QI projects that they could implement themselves and that targeted aspects of their own practices they perceived in need of change. Only two work teams developed projects concerning changes to be implemented elsewhere along the healthcare continuum or by other people within the organization. It was also encouraging to observe that all but one of the action plans targeted the improvement of collaboration and communication among the professionals from different disciplines attending the workshops.

Two action plans developed by the work teams are described below: the first targeted a specific aspect of care, parallel collaborative practice, and was easily implemented immediately after the workshop, while the second was broader in scope, targeted the development of concerted collaborative practice and required implementing changes at various levels.

### A retinopathy screening plan

The work team in one workshop was concerned about the high percentage of retinopathy cases in their subregion. With a view to acting quickly to prevent retinopathies, the professionals on this team opted to improve their screening processes. Through reflection, they ascertained that they did not know whether a member of their healthcare team (physician or nurse) informed a given patient of the need for an eye examination or referred him or her for such an examination; they further recognized that this step may be omitted during their patient consultations. To build this aspect of follow-up systematically into appointments, they developed an action plan for creating a reminder system: a label affixed to the file and on which the professionals must enter the date when a patient is referred for an eye examination and the date when it takes place. This labelling system facilitated communication between physician and nurse regarding this important aspect of diabetic patient follow-up and required few resources. One physician in the family medicine group was appointed project coordinator. Immediately after the workshop, he asked an administrative assistant at the clinic to create eye-shaped labels and to affix them to the files of the clinic’s diabetic patients. All the clinic’s physicians and nurses were informed of this new procedure for improving patient retinopathy screening.

### A project aimed at improved screening for people with diabetes or at risk of developing diabetes

In one subregion, by reflecting on their performance, the members of one work team ascertained that their practices generally adhered to the guidelines. However, they also recognized that they probably took action too late with their diabetic patients, which possibly explained the high frequency of cardiovascular and visual complications and the presence of numerous comorbidities in this population. This team therefore drew up an action plan aimed at developing a screening and early management program for pre-diabetic or at-risk patients. A physician and nurse in one family medicine group were named the project coordinators at the workshop. After reviewing the literature, they developed a program for patients aged 40 and over with blood glucose levels equal to or higher than 5.6. The nurse reviewed these patients’ files for the presence of risk factors. The physicians of patients found to have risk factors were asked for a prescription for a laboratory oral glucose tolerance test. The patients saw their physician again after the test, if needed, to determine whether they were diabetic, pre-diabetic, or at risk of diabetes. At-risk patients were referred to the nurse in the family medicine group for a first educational intervention on risk factors, while pre-diabetic and diabetic patients were referred to their regional diabetes education centre. Project implementation took two years and involved many steps: convincing all physicians in the family medicine group to participate in the project; reviewing files to identify at-risk patients; reaching agreements with the hospital’s laboratory services department to change the protocol for the oral glucose tolerance test to adapt it better to the population’s needs; and establishing collaborative initiatives with the diabetes education centre in order to launch a new intervention for pre-diabetic patients. These steps also required collaboration with the management staff of the health and social services centre (CSSS) to ensure acceptance of the changes and allocation of resources. Lastly, this team developed a data collection system for monitoring the outcomes of their program by tracking the number of persons screened and monitoring the health of the screening-program participants annually.

## Discussion

The aim of this study was to assess if the implemented intervention achieved its expected short term outcomes. Our study results demonstrate that the COMPAS intervention enables front-line professionals to target mutual practice improvement goals and to develop action plans that promote the improvement of IPC and cooperative practice change. Performance feedback and reflective learning promote the definition of a common goal to be attained by participants and the development of an action plan, a shared project for attaining this goal. Clinicians’ involvement in this process is conducive to their developing a commitment to improving the healthcare system. According to Ham [30], what matters most to health professionals is delivering quality services that improve their patients’ health. Strategies designed to promote their involvement in the change process are more likely to engage them than strategies which imply tighter control over practices. Engaging clinicians in the change process is achievable by encouraging them to recognize needs and change priorities, but also by offering them organizational support. As described in the InQuIRE framework developed by Brennan et al. [31], multiple factors influence QI in primary care. This framework describes that contextual factors, at the organizational context, team work and individual levels, act as both antecedents and proximal outcomes of QI in primary care. These factors may be modified by participation in the CQI process and activities. The COMPAS intervention aims to increase capability for continuous QI, readiness to implement process of care changes and team shared goals [24]. However, other factors not presently addressed by the COMPAS intervention, such as available resources, climate and culture for change and leadership, are also important required conditions to successfully implement these practice changes [31].

Improving IPC in primary care is a challenge because of multiple barriers such as definition and awareness of one another’s roles and competences, shared information, confidentiality and responsibility [12, 14]. Providing professionals with feedback and protected time to reflect together and face-to-face on their practice can be beneficial. As described by the results of this study, physicians, nurses and pharmacists are still trying to find ways to improve how they share information and adequately refer patients to appropriate available services. Most of the action plans developed were not yet targeting higher levels of collaboration but we expect that the repeated conduct of the COMPAS intervention for different chronic conditions in the same setting can gradually support the development of more concerted and shared healthcare practice. The population health approach and a focus on the best possible care and services promoted by the COMPAS intervention are important components for the development of effective team functioning which include team processes based on shared objectives, participation, quality and support for innovation [13].

In this project, some individuals and teams succeeded in implementing changes in their practice setting. While we were unable to formally measure the implementation rate of the developed action plans, we know that other groups were unable to implement the planned changes due to lack of time, resources, leadership, or organizational support. Given these results, we revisited certain characteristics of our intervention. To improve impacts, we decided to change the way the workshops will be delivered so as to let the different practice settings take ownership of the continuous QI strategy proposed by the COMPAS project. Instead of using workshop facilitators who go from one setting to another, we therefore hope to train the middle managers currently responsible for chronic disease management in their subregion to play this role. These middle managers will be able to use the COMPAS process to motivate professionals within their organization to participate in and commit to the continuous improvement process. In accordance with the “Model for Improvement” developed by Langley et al. [32], they will be trained to use this intervention at the planning stage of the “plan-do-study-act” cycles and to further support the implementation and evaluation of the developed action plans. According to Birken [33], middle managers work under the supervision of an organization’s senior managers while also supervising and supporting employees, and are thus optimally placed to influence both senior management and clinical practices. Their role should be to offer professionals the means and resources needed to change their practices on a daily basis and to facilitate the adoption of innovations.

The strengths of this study are twofold: it confirms the plausibility of the intervention’s underlying theory and that the intervention’s action mechanisms allow the development of relevant action plans promoting improved management of chronic diseases. One of its limitations is its exploratory nature: it did not demonstrate the effectiveness and superiority of the COMPAS intervention in supporting practice changes over other interventions aimed at QI of services. However, repeated observation of the intervention, data collected on the action plans, and exchanges held with some participants provided the researchers with in-depth knowledge of the intervention’s action mechanisms and allowed coherent changes to be made to the intervention.

Lastly, implementation of the COMPAS intervention is ongoing and workshops on chronic obstructive pulmonary diseases are now being offered. We are currently planning a training program designed for middle managers responsible for chronic diseases, who will soon be giving workshops in their own settings. Furthermore, from then on, interviews with named project leaders during the workshops are conducted to improve our understanding of their role, their capability for change, and factors influencing implementation of action plans in different contexts. Another challenge faced by our team is finding valid and reliable tools to measure the impact of the COMPAS intervention on quality of care, patients’ health and IPC. However, selecting appropriate instruments for QI in primary care is complex because of limited evidence on measurement properties and heterogeneous results between studies [31].

## Conclusion

Our study results demonstrate that the COMPAS intervention enabled professionals to target priorities for practice improvements and to develop action plans that promote IPC. We think that the proposed approach can be very useful to support and engage primary care professionals in the planning stage of QI projects since it combines key successful ingredients: feedback, reflection and planning of action.

## Additional file

Additional file 1:
**Action plan template.** (DOCX 23 kb)

## Abbreviations

IPC: Interprofessional collaboration
CPD: Continuing professional development
QI: Quality improvement
