# Supplementary figures and images for: Combining administrative data feedback, reflection and action planning to engage primary care professionals in quality improvement: qualitative assessment of short term program outcomes
Source: BMC Health Serv Res. 2015 Sep 18;15:391. doi: 10.1186/s12913-015-1056-0 (PMC4574571; doi:10.1186/s12913-015-1056-0)

Additional File 1. Action plan template


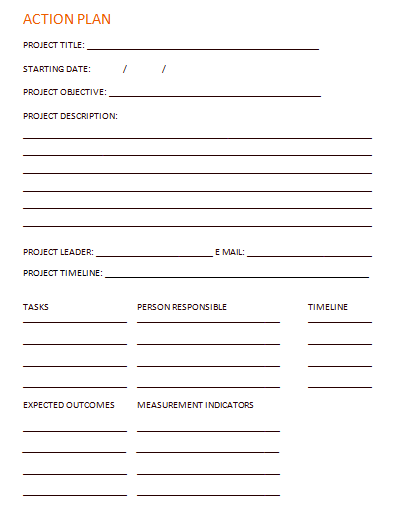

Supplement: Additional file 1: — Action plan template. (DOCX 23 kb) [file 12913_2015_1056_MOESM1_ESM.docx]
